# 3D-printed weight holders design and testing in mouse models of spinal cord injury

**DOI:** 10.3389/fddev.2024.1397056

**Published:** 2024-05-22

**Authors:** Sara De Vincentiis, Francesca Merighi, Peter Blümler, Jose Gustavo De La Ossa Guerra, Mariachiara Di Caprio, Marco Onorati, Marco Mainardi, Vittoria Raffa, Marina Carbone

**Affiliations:** ^1^ Department of Biology, University of Pisa, Pisa, Italy; ^2^ Department of Information Engineering, University of Pisa, Pisa, Italy; ^3^ Department of Physics, Johannes Gutenberg-University Mainz, Mainz, Germany; ^4^ Neuroscience Institute, National Research Council, Pisa, Italy; ^5^ Laboratory of Biology “Bio@SNS”, Scuola Normale Superiore, Pisa, Italy; ^6^ Department of Biomedical Sciences, University of Padua, Padua, Italy; ^7^ e-SPres3D s.r.l., Pisa, Italy

**Keywords:** wearable device, 3D printing, non-invasive stimulation, spinal cord application, external magnetic field

## Abstract

This paper details the comprehensive design and prototyping of a 3D-printed wearable device tailored for mouse models which addresses the need for non-invasive applications in spinal cord studies and therapeutic treatments. Our work was prompted by the increasing demand for wearable devices in preclinical research on freely behaving rodent models of spinal cord injury. We present an innovative solution that employs compliant 3D-printed structures for stable device placement on the backs of both healthy and spinal cord-injured mice. In our trial, the device was represented by two magnets that applied passive magnetic stimulation to the injury site. This device was designed to be combined with the use of magnetic nanoparticles to render neurons or neural cells sensitive to an exogenous magnetic field, resulting in the stimulation of axon growth in response to a pulling force. We show different design iterations, emphasizing the challenges faced and the solutions proposed during the design process. The iterative design process involved multiple phases, from the magnet holder (MH) to the wearable device configurations. The latter included different approaches: a “Fitbit”, “Belt”, “Bib”, and ultimately a “Cape”. Each design iteration was accompanied by a testing protocol involving healthy and injured mice, with qualitative assessments focusing on animal wellbeing. Follow-up lasted for at least 21 consecutive days, thus allowing animal welfare to be accurately monitored. The final Cape design was our best compromise between the need for a thin structure that would not hinder movement and the resistance required to maintain the structure at the correct position while withstanding biting and mechanical stress. The detailed account of the iterative design process and testing procedures provides valuable insights for researchers and practitioners engaged in the development of wearable devices for mice, particularly in the context of spinal cord studies and therapeutic treatments. Finally, in addition to describing the design of a 3D-printed wearable holder, we also outline some general guidelines for the design of wearable devices.

## 1 Introduction

3D printing has revolutionized the manufacture of biomedical devices. This technological advancement is reshaping the landscape of product design and production, presenting a paradigm shift with advantages such as improved flexibility, accelerated production speed, and unparalleled possibilities for customization (Berman, 2012).

The scope of 3D printing in clinical medicine is multifaceted, (Berman, 2012; Prendergast and Burdick, 2020; Kalaskar, 2022), underscoring its vast potential. Beyond its applications in clinical settings, 3D printing has made significant contributions to advancing preclinical research (Goyanes et al., 2018; Wang et al., 2018). In specific scenarios, 3D printing serves as a viable substitute for traditional microfabrication processes (Cho et al., 2017).

Moreover, within the realm of preclinical research, 3D printing stands out as an enabling technology for the development of wearable devices dedicated to animal models, thus emphasizing its versatile impact across diverse scientific domains.

The necessity of implementing wearable devices on mice has become increasingly evident. While various solutions have been proposed in the past for brain applications (Dombeck et al., 2007; Kerr and Nimmerjahn, 2012; Kobayashi et al., 2019; Zhang et al., 2021), the extension of these devices to the spinal cord has emerged only recently, mainly driven by technical and behavioral challenges. One of the primary drivers is linked to imaging the spinal cord in freely behaving mice. In this scenario, methods have been developed to maintain an unrestrained animal beneath a conventional microscope mounted over glass windows or implanted chambers (Farrar et al., 2012; Fenrich et al., 2012; Cheng et al., 2016; Sekiguchi et al., 2016). Despite providing precise control over sensory input and motor response readouts, these methods have some weaknesses (Nelson et al., 2019), including impractical long-term use. Consequently, alternative approaches have been suggested that employ miniaturized implanted microscopes adapted for use on unrestrained animals which facilitate repeated measurements over consecutive days (Sekiguchi et al., 2016; Nelson et al., 2019; Shekhtmeyster et al., 2023).

Optoelectronic and wireless optogenetic systems have also been implanted (Montgomery et al., 2015; Park et al., 2015; Wang et al., 2020), but the oxidation of grafted stimulation wires limit their reliability (Kathe et al., 2022). Other interesting applications of wearable devices are represented by the possibility of conducting chronic treatments on the dorsal region of mouse models, with special regard to the spinal cord. For example, Yao et al. (2019) sutured a device for therapeutic electrostimulation onto the backs of mice, promoting hair regeneration. This region was also chosen for subcutaneously placing devices for the automatic release of drugs, as exemplified in the case of naloxone, which is used as an antidote in cases of overdose (Dhowan et al., 2019).

In the context of the different wearable devices mentioned above, their fixation involves at least the use of surgical sutures or, in more invasive cases, partial or total subcutaneous implants. In addition to the inherent constraints associated with spinal cord implants—mechanical issues and limited biointegration due to their dynamic nature (Montgomery et al., 2016) —more limitations arise from the invasiveness of surgical procedures, particularly on mice subjected to experimental lesions. Consequently, our study aimed to administer the stimulus of interest—a constant static magnetic field—externally rather than through an internally implanted device. To achieve this, compliant 3D-printed structures were designed for mice for use in both healthy and spinal cord-injured subjects allowing secure placement of magnets at the target site.

Very few studies have used externally applied devices and, to our knowledge, none for treatments on the spinal cord. Concerning the necessity of securing materials onto the backs of small rodents, a similar approach has been developed for cerebral treatments requiring a wearable system by freely moving rats (Kim et al., 2021; Kim et al., 2022). With entirely different objectives, the Belgian non-profit organization APOPO has equipped rats with neoprene backpacks containing a 3D-printed plastic container housing a video camera. This initiative aims to aid first responders in locating survivors amidst rubble in disaster zones (La Londe et al., 2015; Patrikakis, 2023). Regardless of the purpose, the backpack is the most widely used design, with a preference for rats. While reducing both size and weight remains a critical consideration in engineering designs for long-term studies across all scenarios, rats have proven able to withstand heavier loads than mice. Reports indicate that well-tolerated weights in adult rats range from 20 g to 35 g (Kim et al., 2021; Kim et al., 2022), whereas mice can hold their own weight (Deacon, 2013), even if Shekhtmeyster et al. (2023) suggests that 8- to 16-week-old mice can comfortably carry approximately 10 g following some habituation.

We sought to create a device that could be primarily used in mouse models. This required a special focus on weight and size reduction while maintaining adequate mechanical resistance for the long-term durability required by full-time application on freely moving mice kept in standard rearing conditions—in home cages containing at least three individuals.

The process by which mechanical force promotes axon growth is termed “stretch growth” (Smith, 2009). We describe our experience in designing and prototyping a 3D wearable device for mice, reporting the challenges faced and the solutions proposed in the context of a project that aimed to test the *in vivo* therapeutic potential of stretch growth to stimulate and promote *in situ* survival, differentiation, and the integration of neuronal precursors loaded with magnetic nanoparticles engrafted into the injured spinal cord. We had previously demonstrated that the *in vitro* exposure of neurons loaded with nanoparticles to a magnetic field generates mechanical forces that can enhance the elongation of neurites (Raffa et al., 2018; De Vincentiis et al., 2020).

To test the *in vivo* efficacy of stretch growth, we needed to develop a solution allowing both healthy and injured mice to carry magnets with a size and shape determined to generate a magnetic field with proper orientation and intensity. In addition, it was crucial for the stimulation to be continuous in time (Raffa et al., 2018). Therefore, the mice needed to wear the magnets continuously throughout the duration of the stimulation, estimated to be at least 21 days.

We were able to fulfill these requirements by minimizing the weight of a 3D-printed non-deformable structure that was essential to ensuring the steady and continuous positioning of a couple of magnets for long-term application of the desired and previously optimized (Riggio et al., 2014; De Vincentiis et al., 2023) magnetic field to the spinal cord of mice.

## 2 Materials and methods

### 2.1 Ethical statements

All experiments were performed in accordance with the approved guidelines of the Italian Ministry of Public Health local Ethical Committees and Directive 2010/63/EU.

Mouse hippocampal neurons were isolated in the animal facility (authorization for animal breeding n° 1,695 of 12/10/2023 from Comune di Pisa) located at University of Pisa, Department of Biology, Unit of Cell and Developmental Biology I S.S. 12 Abetone and Brennero 4 (license number 39E1C.N.5Q7 of 30/10/2021).

The animals for *in vivo* trials were housed in the animal facility of the CNR Neuroscience Institute, and experiments were authorized by the Italian Ministry of Health document 647/2022-PR.

In both cases, C57BL/6J mice were housed under controlled conditions, maintaining a temperature of 23°C ± 1 °C, humidity 50% ± 5%, and a 12-hour light–dark cycle. Mice were given unrestricted access to both food and water.

Special care was taken to monitor the status of discomfort, stress, and pain of mice enrolled, and, in cases of excessive distress, animals were excluded from further testing and, where no recovery could be observed, humane endpoints were adopted.

### 2.2 Cell isolation and magnetic nano-pulling protocol

For hippocampal neurons, neonatal animals at postnatal day 1 were euthanized, and both hippocampi were collected in a solution containing D-glucose at a 6.5 mg/mL concentration in DPBS (Gibco, Thermo Fisher Scientific, Waltham, Massachusetts, US, #14190-094). Cell isolation was achieved through a combination of chemical digestion and mechanical dissociation, following De Vincentiis et al. (2020) and Falconieri et al. (2023). The cells were then cultured in high-glucose DMEM (Gibco, Thermo Fisher Scientific, Waltham, Massachusetts, US, #21063-029) supplemented with 10% fetal bovine serum (FBS, Gibco; Thermo Fisher Scientific, Waltham, Massachusetts, US, #10270-106), 100 IU/mL penicillin, 100 μg/mL streptomycin (Gibco, Thermo Fisher Scientific, Waltham, Massachusetts, US, #15140-122), and 2 mM Glutamax (Gibco, Thermo Fisher Scientific, Waltham, Massachusetts, US, #35050-038). Cells were seeded onto 2-well chambers (Ibidi, Gräfelfing, Germany, #80106) at a density of 150,000 cells/chamber. The culture surfaces were pre-coated with 100 μg/mL poly-L-lysine (PLL, Sigma-Aldrich, Burlington, Massachusetts, US, #P4707) and 10 μg/mL laminin (Sigma-Aldrich, Burlington, Massachusetts, US, #L2020). Four hours later, the medium was replaced with a cell-culture medium comprising Neurobasal-A medium (Gibco, Thermo Fisher Scientific, Waltham, Massachusetts, US, #12348-017) modified with B27 (Gibco, Thermo Fisher Scientific, Waltham, Massachusetts, US, #17504-044), 2 mM Glutamax (Gibco, Thermo Fisher Scientific, Waltham, Massachusetts, US, #35050-038), 50 IU/mL penicillin, and 50 μg/mL streptomycin. Superparamagnetic nanoparticles (FeraSpin™ XL MRI agent, #130–095-172, Viscover, Berlin, Germany) were added to the medium at 100 μM concentration in both control and stimulated groups. The FeraSpin™ nanoparticles have a hydrodynamic diameter of 50–60 nm; these are multi core particles with an effective magnetic moment of *m*_eff_ = 1.6 · 10^−17^ Am^2^ per nanoparticle and a saturation magnetization of *M*_s_ = 335 kA/m (Kahmann and Ludwig, 2020). From the next day, the magnetic device was applied continuously for 48 h to the “stretch” group.

### 2.3 Requirements

The wearable device had to meet several key criteria:

1. Magnet configuration and weight: the device should house two magnets of predetermined dimension and weight inserted in a fixed relative configuration.
2. Positioning of magnets: magnets should be placed on the mouse’s back to maintain a predefined distance (3.3 ± 0.5 mm) between the magnets’ inferior surface and the injury site, at a distance determined by the thickness of the overlying tissues (muscles and skin).
3. Duration of wear: the device should be worn by the mice for at least 3 weeks.
4. Magnetic field inversion: ideally, the magnets’ block should be rotated 180° every 24 h to allow magnetic field inversion.
5. Control mice: these should not be equipped with magnets but should wear an identical device with matching weight characteristics.

In addition to project specifications, the design process carefully considered findings from existing literature, as well as the authors’ experience. These supplementary factors, which were crucial for the design, included considerations such as weight tolerance, ensuring that the wearable device adhered to a stringent weight limit relative to the mouse’s body mass (Deacon, 2013) or even less (Shekhtmeyster et al., 2023). Mobility requirements were also taken into account to allow the free movement of mouse forelimbs, essential for maintaining proper grooming and reaching food (Kalueff et al., 2016). Furthermore, measures were implemented to control stress levels, ensuring that the wearable device did not unduly induce stress or depression and so safeguarding animal welfare and the clinical trial from potential behavioral bias (Du Preez et al., 2020; Du Preez et al., 2021).

### 2.4 Magnets configuration

**FIGURE 1:**
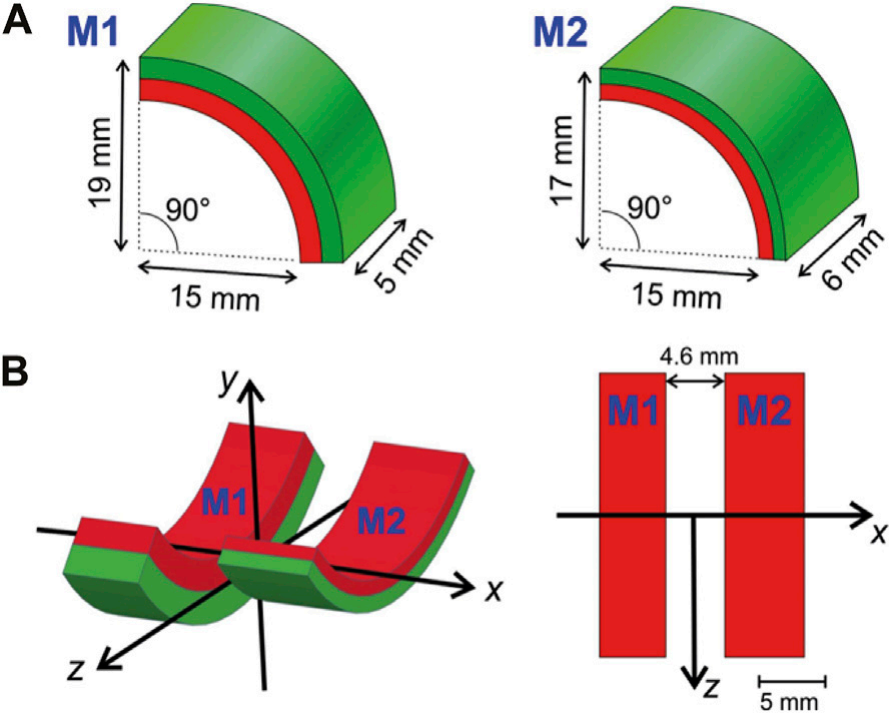
Magnets. **(A)** The magnetic applicator consists of two radially magnetized permanent magnet quarter-hollow cylinders (arcs) of slightly different geometry. One arc (M1) is thicker (5 mm vs. 2 mm) but shorter than the other (M2). **(B)** Both arcs are arranged with a gap of 4.6 mm between them. The left graph shows the coordinate system used in a 3D view, while the right is a projection of the arrangement from the top of the *y*-axis. The device is mounted dorsally (*i.e.*, on the back of a mouse) such that the positive *y*-axis points ventrally (towards the abdomen) and the *x*-axis points parallel to the spinal cord.

In order to generate a strong magnetic force on nanoparticles along the direction of the spine—defined as the *x*-axis (Figure 1A–B)—the magnet system must produce a strong magnetic field gradient in this direction. This was readily achieved by placing two magnets in antiparallel position (Figure 1B). The magnetic force $\vec{F}$ on a superparamagnetic object is given by

$$\vec{F}=\vec{\nabla }\;\left(\vec{m}∙\vec{B}\right),$$ (1)

which is the spatial derivative (or divergence) $\vec{\nabla }=\left(\frac{\partial }{\partial x},\frac{\partial }{\partial y},\frac{\partial }{\partial z}\right)$ acting on the magnetic moment $\vec{m}$ of the nanoparticle in an external magnetic field, $\vec{B}$ . The magnetic moment, on the other hand, is strongly dependent on the magnetic field, which is typically given by the Langevin-function, L

$$m\left(B\right)=m_{s}\;L\left(\xi \right)\approx {\;M}_{s}V\;L\left(\xi \right)\text{ with }L\left(\xi \right)=\coth\xi \;-\frac{1}{\xi }\text{ and }\xi =\frac{M_{s}V}{k_{B}T}B,$$ (2)

where *V* is the volume of the particle, *k*_B_ is the Boltzmann constant, and *T* is the absolute temperature. Hence, *m* is zero and so the force is absent of a magnetic field, as would be the case in the center between two identical antiparallel magnets. Therefore, a slightly asymmetrical arrangement was chosen (Figure 1A). Both magnets were made of FeNdB N52 (remanence ca. 1.45 T) and were nickel-coated (Spacemagnets GmbH, Hürth, Germany).

**FIGURE 2:**
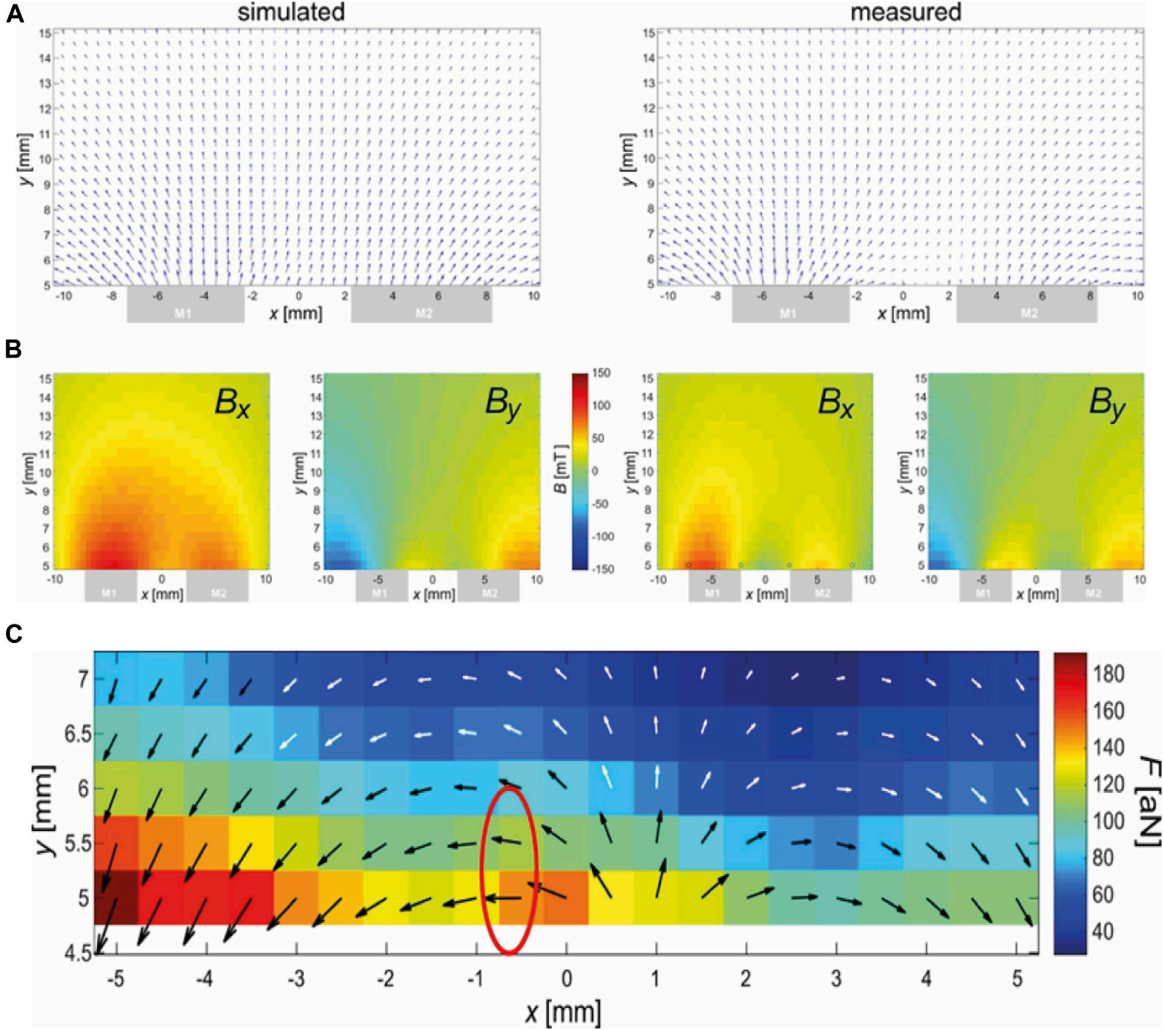
Magnetic field **(A, B)** and force **(C)** of the magnetic arrangement. **(A)** FEM simulation of the magnetic field. **(B)** Measurement of the magnetic field. Central *xy*-slice (*z* = 0) shown. Top: Vectorial depiction of *B*_*x*_ (*x*,*y*) and *B*_*y*_(*x*,*y*), which are shown with an identical scale below. The gray boxes at the lower sides indicate the position of the magnets in the *x*-direction. Note that the *y*-direction does not start at the surface (0 mm) of the magnets due to an unavoidable mechanical offset of *y* = 4.5 mm caused by the mounted 3D-Hall-probe. **(C)** Calculated magnetic force using data from **(B)** and Eq. 3. The black-and-white arrows illustrate the direction of the force while the underlying colors relate this length to the amplitude of the force in attonewtons (10^−18^ N). The red encircled region was chosen for placement at the spot of the lesion because here the force is strong and horizontally (*i.e.*, in the direction of the spine).

This magnet arrangement was chosen based on simulated (COMSOL Multiphysics, version 6.1) field data. The real arrangement was measured using a 3D Hall-probe (TLV493D, Infineon) positioned via a home-built 3D-stage. The results of the finite-element simulations (FEM) and the measurement are compared in Figures 2A, B. The agreement is reasonable considering easy misplacements of magnets and sensors at such small scales additional to the general variations (typically in the percent range) of the properties of permanent magnet materials.

To estimate the local force, Eqs 1, 2 were combined. However, the direction of $\vec{m}$ of the nanoparticles will orient in the direction of the local magnetic field $\vec{B}$ , and the dot-product in Eq. 1 can be replaced by the product of the magnitudes because both vectors are parallel. Consequently, the combination of Eqs. 1, 2 gives (Blümler et al., 2024)

$$\begin{array}{l} \vec{F}=\vec{\nabla }\left(mB\right)=\vec{\nabla }\left(M_{s}V\;L\left(\xi \right)B\right)=M_{s}V\;L\left(\xi \right)\vec{\nabla }B+{BM}_{s}V\;\vec{\nabla }L\left(\xi \right)=M_{s}V\left(L\left(\xi \right)+\xi \frac{dL}{d\xi }\right)\;\vec{\nabla }B={\;M}_{s}V\;P\left(\xi \right)\;\left(\frac{\partial B}{\partial x},\frac{\partial B}{\partial y},\frac{\partial B}{\partial z}\right)\\ \text{with }P\left(\xi \right)=\coth\xi -\xi {\mathrm{csch}}^{2}\xi \text{ and }B=\sqrt{B_{x}^{2}+B_{y}^{2}+B_{z}^{2}}. \end{array}$$ (3)

Figure 2C shows the calculated force based on the measured data from Figure 2B and the application of Eq. 3 in a relevant region (expected position of murine spine). It must be emphasized that Eq. 3 refers to the magnetic force exerted on a single nanoparticle, while there is strong clustering observed, especially in the presence of magnetic fields, due to mutual dipolar forces. Depending on the shape of the cluster, the force can be up to *n*-times that of Eq. 3 for *n* particles in the cluster (Blümler, 2021).

Figure 2C shows an encircled region where the force is parallel to the spinal cord in approximately the correct distance to the magnet. This position should be placed at the position of the lesion. Here, the following parameters were estimated: *B*_*x*_ = 27 mT, *B*_*y*_ = −1 mT, $\frac{\partial B}{\partial x}$ = −8 T/m, $\frac{\partial B}{\partial y}$ = −0.07 T/m, *F*_*x*_ = 146 aN, *F*_*y*_ = −1 aN, ξ = 101, $P\left(\xi \right)$ = 1.

### 2.5 Magnet holder (MH) design

The first step was to design the magnet holder (MH). Considering the magnet weight (6.38 ± 0.25 g), dimensions, relative position, and repelling forces generated by the side-by-side arrangement (Figure 1B), a holder was designed to keep them firmly in place. CAD software (PTC Creo, version 8.0, https://www.ptc.com/it) was used for computer-aided design throughout the whole process.

The MH design was fabricated using a PolyJet™ 3D printing system—the Objet M30 3D printer developed by Stratasys Inc. (Stratasys, Inc. IS). The selected material for this construction was MED610 resin, a biocompatible PolyJet™ material specifically approved for bodily contact. The material is designed to provide greater efficiency and more cost-effective productivity across a range of medical and dental applications. Biocompatible MED610 is suitable for permanent (more than 30 days) contact with intact skin, as well as non-permanent contact with mucosal membranes, breached or compromised surfaces, tissues, and bones (Stratasys Biocompatible MED610 Stratasys, 2024). Given these attributes, the material was deemed suitable for interface with injured mouse skin.

The removal of supports from specimens constructed with the medical-grade material MED610 adhered to the “Bio-Compatibility Requirements” guidelines provided by the material supplier, ensuring compatibility with biological systems.

The holder was modified for each design via fixation on mice.

**FIGURE 3:**
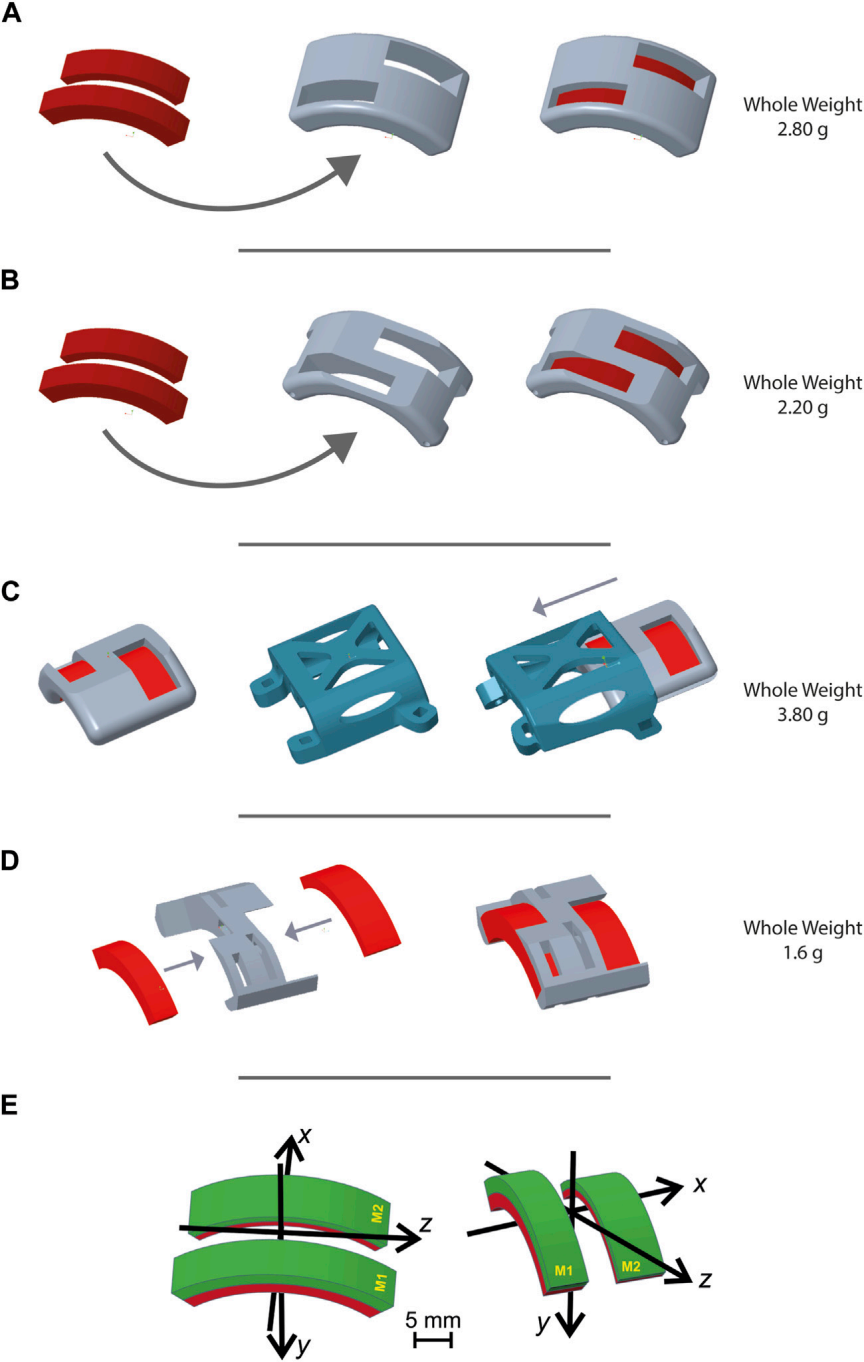
Magnet holder designs. Figure 3 shows representative images generated with PTC Creo Parametric 8.0 -PTC software of different magnet holder configurations. Through an iterative design process, the MH evolved over time to be optimized according to the requirements established for the design. **(A)** First MH design presented two holes for the two magnets, designed to facilitate their insertion, while also reducing the device weight. **(B)** Second MH design based on the previous one but with two main modifications: a cut of the superior surface to render the entire structure lighter, and the second on the short sides to be associable with the second design iteration. **(C)** Third MH design: in order to accomplish the requirement of inverting the direction of the magnet, this third design was composed of a stable element, the slide, and a removable and invertible MH. The slide was designed as lightly as possible and with a hookup necessary to secure it into the third design iteration. **(D)** Fourth MH design fulfills the lightness requirement much better. The two long sides were eliminated and the addition of more holes on the superior surface made this MH the lightest compared to those previously shown. This design required the use of cyanoacrylate glue to ensure a stable fixation of the magnets in their slots. This fourth MH was designed for its use in the design iteration n. 4. **(E)** Coordinate systems and magnet positions for **(A, B)** on the left and **(C, D)** on the right.

Design iteration no. 1 (Figure 3A) underwent a weight adjustment process subsequent to the initial trial: the design was refined to achieve a lighter configuration, resulting in a weight reduction of 2.80 g to 2.20 g (Figure 3B). This simplification did not compromise the ability of the MH to securely and robustly maintain magnets in the intended configuration. Moreover, changes were performed in the short sides of the holder to improve fixation to the animal’s body in design iteration no. 2.

To make periodic inversions of the magnetic field orientation (*i.e.*, 180° flipping of both magnets along their main axis), a slide approach was designed for the holder (Figure 3C). The slide was designed to combine light weight with stable positioning on the mouse’s back, while the MH would be the movable element for magnetic field inversion. The ensuing design iteration no. 3 had a total weight (slide plus) of 3.80 g.

This solution, even if apparently suitable according to our initial tests on healthy subjects, was then abandoned as it turned out to be too heavy for the injured mice (see “Results” section for details).

In pursuing an optimal solution, the ultimate trial led to a further reduction in weight, culminating in the definitive final design (Figure 3D). This design (weight 1.6 g) lacked the mechanical resistance required to hold the two magnets in place. However, this was easily solved by applying 0.2 g of cyanoacrylate inside each magnet slot.

Additionally, the lack of direct contact between the final design of the MH element and the surgical wound allowed us to print it using ABS M30 material from Stratasys, employing a fused deposition modeling (FDM) printer (Fortus F170, Stratasys INC). This lighter material allowed the final device to reach a weight of only 0.80 g.

For each trial, control mice (*i.e.*, not be exposed to the magnetic field) received an identical MH, containing identically weighing mock magnets fabricated from lead.

### 2.6 Wearable device design iterations

#### 2.6.1 First design: “Fitbit” approach

**FIGURE 4:**
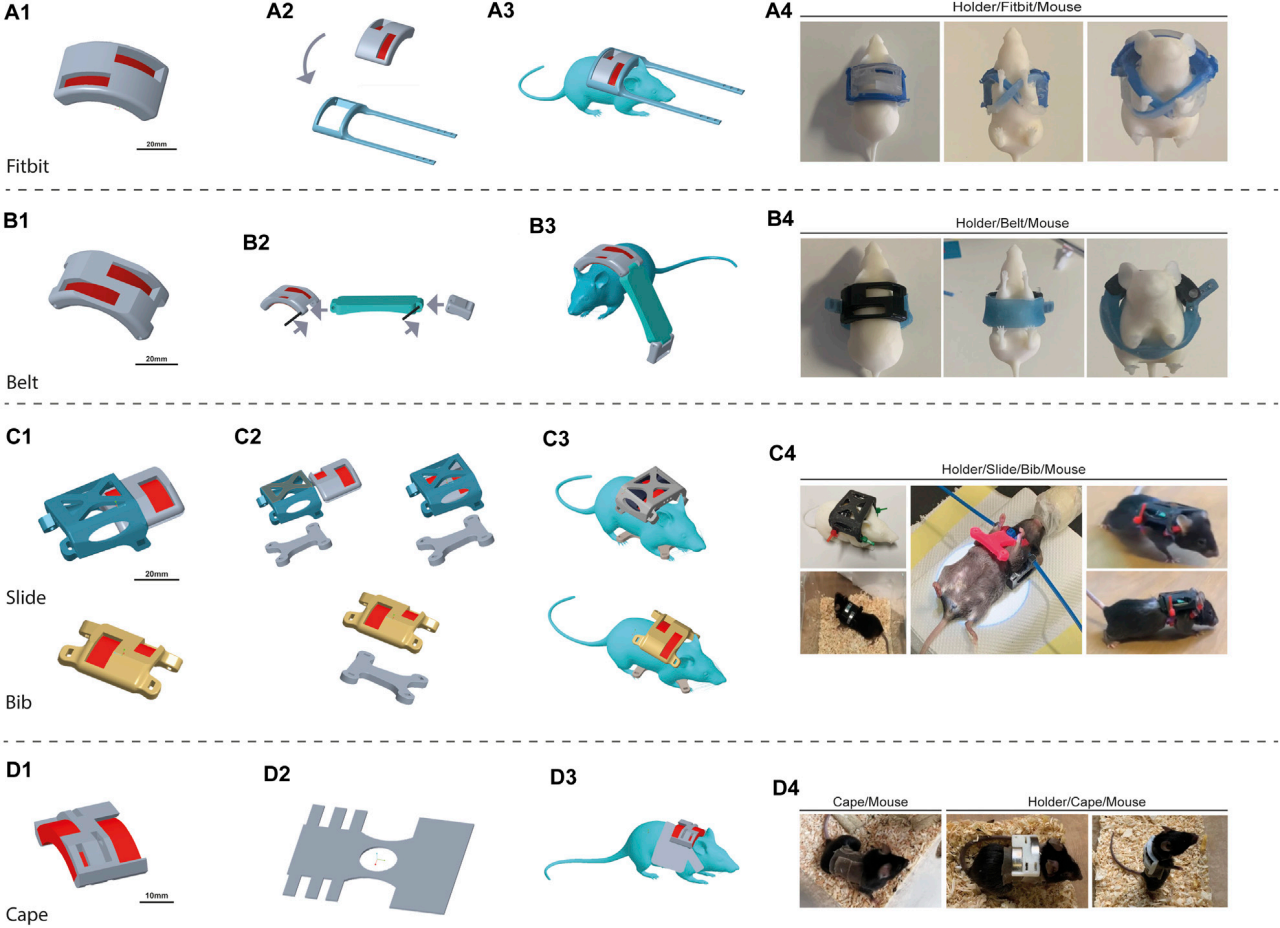
Design iterations. The device is composed of the 3D-printed magnet holder (MH) and the wearable structures, generated in silicone. We faced challenges in each step, and thus, we proposed different solutions to overcome the issues gradually encountered. The proposed configuration includes different approaches: “Fitbit” **(A)**, “Belt” **(B)**, “Bib” **(C)**, and ultimately “Cape” **(D)**. The “Bib” presented two variations: “Slide Bib” and “Simple Bib”. For each design is shown the 3D render image of the project of the magnet holder (1), of the complete wearable structure with the silicone component (2), and of the mice wearing the device. Four pictures show the real and produced devices. For “Fibit” and “Belt,” a 3D printed mouse was dressed to give an example of the wearability. No pictures are available on real mice because, after dressing, they almost immediately did not keep the device on. For the “Slide”, “Bib” and “Cape”, a picture of the wearability on real mice is shown.

The first concept was based on designing a silicone-based “Fitbit” band as the foundational element (Figure 4A). This design involved a wraparound component for the MH accompanied by two suspenders that would intersect beneath the animal’s belly and reattach to the initial component. Starting from the MH, a Fitbit band was designed to envelope it and to allow its possible rotation. Then, as the Fitbit band was to be produced in silicone, its corresponding molds were designed as well (Supplementary Figure S1). The molds were 3D printed with the same printer but, since biocompatibility was not required, we selected a different material: VEROClear resin, hence referred to as “the mold material”. Two distinct RTV (room temperature vulcanizing) silicone types underwent testing: DragonSkin™ FX-Pro and Smooth-Sil™ 940, both sourced from Smooth on Inc. (Macungie, PA 18062). The former was swiftly discarded due to its low shore-A value (2A), rendering it excessively elastic and incapable of maintaining the MH securely in place. In contrast, Smooth-Sil, boasting a higher shore-A rating (40A), demonstrated superior compliance in supporting the MH. However, when tested on mice, it became evident that the animals could dislodge it very easily.

#### 2.6.2 Second design: “Belt” approach

In response to these challenges, a second approach was proposed: the “Belt”, where we opted for simplicity by adopting a belt structure. The objective was to create a belt that would represent a compromise between rigidity—thus preventing the mouse easily removal it from its own body—and compliance, to maintain proper breathing and swallowing. The Belt was designed with some rigid (3D printed) parts and a soft (silicone-based) strap. Silicone-based components were again designed together with their molds. The molds were 3D printed with the mold material (Supplementary Figure S2), and the belt was fabricated by pouring silicone into the molds (Figure 4B). This shift in design philosophy aimed to overcome issues of the previous Fitbit design by optimizing the Belt’s fixation on the mouse. By integrating both rigid and compliant elements, the design was supposed to offer the necessary elasticity for proper breathing while being tight enough to prevent removal. Rigid components were printed in MED610, while silicone-based components were molded with DragonSkin ™ FX-Pro. The MH was adapted, as already described in Figure 3B, in order to both decrease its weight and enable the fixation of the silicone components of the Belt. Velcro strips were used to close the Belt. During the trial, it became evident that the Belt was still too easy to remove. Furthermore, the MH was still a very heavy structure (see “Results” for more details).

#### 2.6.3 Third design: “Bib” approach

For this design, the MH was lightened (Figure 3C). The Belt attempt allowed us to understand that the most important aspect of the design was to ensure a stable positioning of the MH without hampering the animal’s ability to breathe. The compliant component was thus considered of utmost importance; however, the recurrent problem of easy undressing emerged again. The subsequent design iteration was focused on preserving a rigid component for the MH while introducing a fundamental alteration in the soft component. As a transition from the Belt configuration, we adopted a “Bib” approach, with the strategy of wrapping the front legs to establish more secure attachment.

The Bib design was prepared in a CAD environment offering variations in size (S, M, and L) to accommodate differently-sized mice; each size maintained a consistent thickness of 2 mm, featuring openings at the extremities of the anterior suspenders to facilitate attachment to the MH. A mold was designed for each of the three sizes of the Bib and was 3D printed with the mold material (Supplementary Figure S3). The chosen molding material, Ecoflex™ RTV silicone from Smooth On Inc., was selected for its exceptionally low shore-A value (00–10) and inherent durability. The molding process comprised three distinct stages:

i) A small amount of silicone was poured into the mold.
ii) Four copper rings and 1.5 × 1 cm^2^ acrylic tissue net were added in the mold. The copper rings were strategically positioned to reinforce the arm apertures, ensuring structural integrity. Simultaneously, the acrylic tissue net served the dual purpose of reducing elasticity and enhancing resistance to potential biting incidents.
iii) Final pour of the remaining silicone.

This procedure aimed to create a resilient and adaptable bib component tailored to different mouse sizes while ensuring durability and effectiveness in maintaining the stability of the MH.

The Bib was fixed to the MH through cable ties which were intentionally configured to ensure complete entanglement. This configuration was employed to eliminate the introduction of a rigid element that could facilitate the mouse's escape. The requirement for the mouse’s movement was ensured by the flexible silicone arms of the Bib. During this iteration, the MH slide was also tested. We thus essentially had two Bib designs: “Slide Bib” (Figure 4C–Slide) and “Simple Bib” (Figure 4C–Bib).

#### 2.6.4 Fourth design: the “Cape”

Given that the results of the third iteration trial were not entirely satisfactory for spinal cord-injured mice (refer to the next section for details), we opted for higher tolerance on certain requirements. Specifically, considering the magnet configuration and mouse mobility, requirement number 2 regarding the distance between the magnet surface and the lesion site was made less stringent. This adjustment permitted the insertion of a tissue of less than 1 mm thickness between the magnet surface and the mouse skin. With these less stringent requirements in mind, the new design iteration was to fix the MH to a “Cape” made of silicone with an incorporated acrylic elastic net (Figure 4D). The rationale behind this modification is rooted in the recognition that a limitation of the Bib approach was its thickness, which hampered grooming movements. Hence, a thinner structure was deemed necessary. However, a thin silicone bib alone lacked the required resistance to biting and mechanical stress. Consequently, the decision was made to enlarge the silicone surface while simultaneously thinning it. The resultant design was referred to as the “Cape”. The Capes were crafted from a single large acrylic net tissue covered with a layer of silicone, with an overall thickness of 0.3 ± 0.2 mm. A cutting template was designed and 3D-printed to ensure repeatability. The Cape was tightened to the mouse by bonding the lateral bands with cyanoacrylate. Initially, the MH was glued directly to the back of the mouse on the cape. However, in further experiments, a modified slide approach will be used to secure its position and facilitate rotation for magnetic field inversion.

#### 2.6.5 Dressing protocol

To minimize stress and manipulation of the animals during the application of the devices, total anesthesia was administered using inhaled isoflurane (4% for induction, 1.5% for maintenance). The rapid metabolism of mice allowed a fast recovery from anesthesia. This choice also complied with ethical considerations, further underscoring our commitment to designing a protocol with special care of the animal’s wellbeing. Once anesthesia was established, the prototype was mounted on the mouse by an expert operator trained and constantly supported by the designers. After recovery from anesthesia, the animal was subjected to a 60-min continuous observation to check its reaction to the device. The animal’s initial mobility and ability to become confident with the weight and footprint of the device, as well as its attempts to remove the device, were monitored. In case the device significantly hindered forelimb mobility, grooming, or food reach, or if the animal performed repeated attempts to free itself from the device, it was immediately removed.

### 2.7 Testing

The testing of wearable prototypes aimed at refining the design of the applicator, it was conducted in parallel with the clinical trial as a measure of ethical refinement aimed at reducing the invasiveness of device application, enhancing survival, and maximizing the effectiveness of passive magnetic field exposure. The latter is essential for achieving the study's primary outcome. The results of continuously evaluating each prototype steered the iterative improvement procedure described in the methods section.

In particular, we determined the duration of time the animal was able to wear the device. A given design iteration was considered successful if worn for 21 consecutive days after injury. The animal was immediately undressed if negative effects or signs of potential device-induced suffering were observed during these 21 days.

All animals were kept in cages containing at least two subjects to facilitate social interaction.

#### 2.7.1 Testing protocol

A crucial habituation period of 7 days was implemented on healthy subjects before the experimental spinal cord injury procedure to alleviate stress induced by the wearable magnetic device.

This period was considered essential to enhancing tolerance of the device. After the injury, a 9-day recovery period was allowed to the animals before starting the magnetic stimulation—that is, device positioning.

This strategy combined the ethical aspects of animal wellbeing with the possibility of evaluating wearable device prototypes on healthy mice prior to testing on injured subjects.

For the last trial (third cohort trial with the Cape design), the habituation period was further improved. For the first 72 h, the mice were gradually introduced to the Cape without the imposition of additional stress from magnet weights. Subsequently, the magnets were inserted into the device (without the need for additional anesthesia) and the mice spent an additional 96 h with the MH mounted. Only after this period of familiarization mice underwent the injury step. This sequence allowed them to gradually habituate to the device, thus minimizing stress.

**FIGURE 5:**
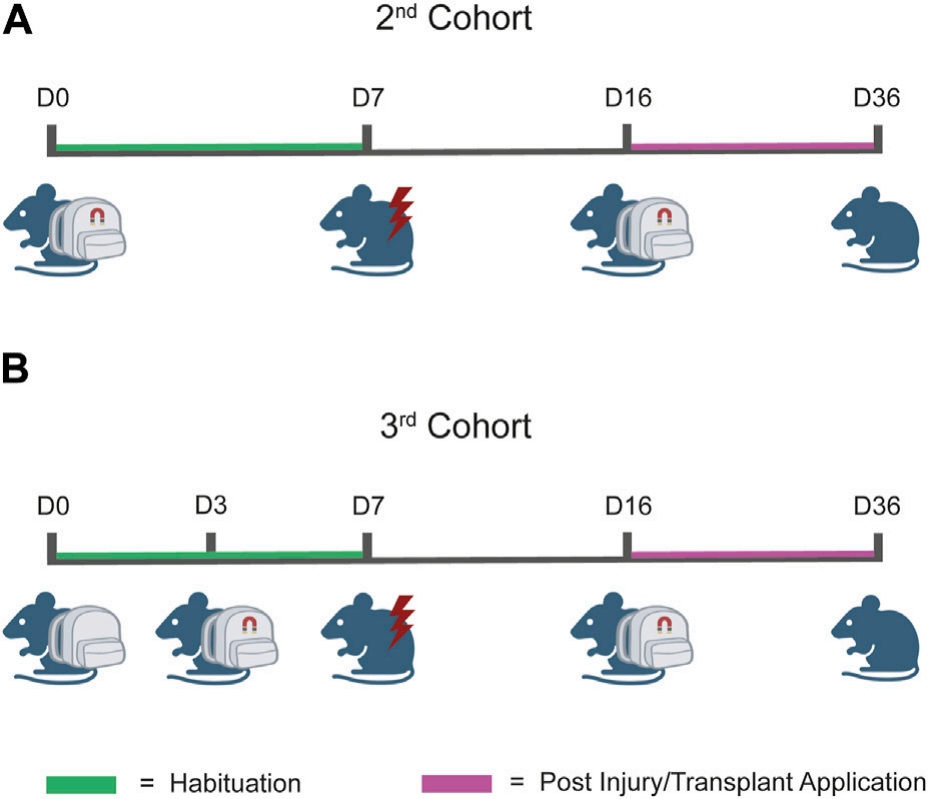
Workflow of the testing protocol. **(A)** Testing protocol for the second cohort. The prototype was composed of the wearable device and magnets which together were applied at day 0 (D0) and maintained until D7, coherently with the designated habituation phase. At D7, the injury was performed, and at D14, cells were transplanted. After 2 days of recovery (D16), prototypes were reapplied to the injured animal to induce magnetic stimulation. **(B)** Testing protocol for the third cohort. The protocol workflow is identical to that illustrated in **(A)** for the second cohort, except for the application time-point of the magnets. In this case, we applied the wearable device at D0 and the magnets were applied at D3. This “split habituation” was established to allow the mice to gradually familiarize with all the components of the applied prototypes.

A schema of the experimental timeline can be found in Figure 5.

### 2.8 Statistical analysis

Analysis of the elongation was performed using image analysis software ImageJ. Neurite length was evaluated using the plugin NeuronJ (Popko et al., 2009), and 100 non-interconnected axons were analyzed from 10× magnification images with a cut-off of 40 µm. Data were plotted and analyzed by Mann–Whitney for non-parametric data with the following equation:

$$U=R_{1}-\frac{n_{1}\left(n_{1}+1\right)}{2},$$ (4)

where *U* is the Mann–Whitney U statistic, *R*_1_ is the sum of the ranks in the first group, and *n*_1_ is the number of observations in the first group. GraphPad software version 6.0 was used for the analysis. Significance was set at *p* < 0.05.

A Kaplan–Meier (Altman, 1990) survival analysis was conducted where we extended the indications of the use of the Kaplan–Meier estimator to determine the maintenance of each tested prototype upon time— the recorded event being the “undress” event. The Kaplan–Meier estimator was calculated using the following equation:

$$S\left(t\right)=\prod_{t_{i}\leq t}\left(1-\frac{d_{i}}{n_{i}}\right),$$ (5)

where *S*(*t*) is the estimated survival probability at time *t*, *t*_i_ are the distinct event times, *d*_i_ are the number of events at time *t*_i_, and *n*_i_ is the number of subjects at risk just before time *t*_i_.

Comparisons between prototypes (groups) were performed using the log-rank test with the following equation:

$$\chi^{2}=\frac{{\left(O-E\right)}^{2}}{E},$$ (6)

where χ^2^ is the test statistic, *O* is the observed number of events, and *E* is the expected number of events.

Prototypes (groups) were compared with the log-rank test. We considered a two-sided *p*-value of less than 0.05 to be statistically significant.

## 3 Results

This section reports all trials conducted during the iterative design phase, focusing on the last iteration, which met all the requirements listed in Section 2.3.

Three trials have thus far been completed.

### 3.1 Magnet configuration and holder design efficacy

**FIGURE 6:**
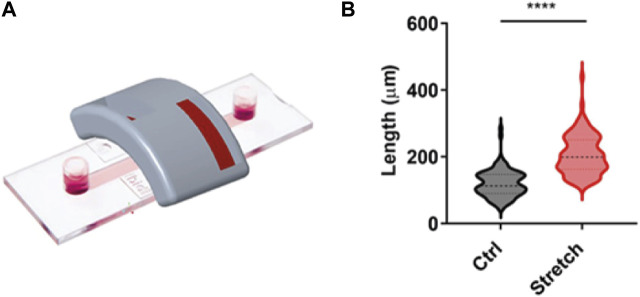
Validation of magnet configuration. **(A)** IBIDI two-well chamber mimicking the distance and orientation of the spinal cord in an *in vivo* context. This configuration was employed for *in vitro* validation of the magnetic support. **(B)** Blind analysis of the axonal length of mouse hippocampal neurons in the setup shown in **(A)**. Violin plot from minimum to maximum, *n* = 100 from four replicates. Mann–Whitney test, *p* < 0.0001.

In order to test the capacity of the magnetic applicator to promote axonal growth, we used our previously established magnetic nano-pulling (Raffa et al., 2018; De Vincentiis et al., 2020; Falconieri et al., 2023). In particular, mouse hippocampal neurons were seeded on 2-well IBIDI chambers (Figure 6A). This device was chosen to maintain the cells at a distance from the magnet comparable to that found *in vivo* between the spinal cord and the magnets. The data (Figure 6B) show a >100% increase in length in the stretched samples compared to the controls. This result represents the maximum value in terms of stretching rate ever achieved by our team in the last 10 years, documenting the effectiveness of the optimization study for the magnetic field gradient and holder design.

### 3.2 First cohort magnetic trial

During the first cohort trial, the first two designs (Fitbit and Belt) were tested.

We started with the Fitbit. The dressing was very tricky and difficult, and the mice immediately got rid of the prototype. Consequently, we decided to abandon the Fitbit approach and abandon dressing the mice. Figure 4A shows the hypothesized device mounting on the mouse. In Supplementary Figure S4, the fabricated components are detailed.

The Belt design was tested as second strategy. We dressed ten mice (mean animal weight (MAW) 20.4 ± 2.81 g); all kept the device on for less than 15 min, being able to easily remove it. In particular, having the forearm completely free (Figure 4B) allowed the animals to push the belt down, even if strongly secured. Further tightening to avoid undressing was not possible due to the risk of inducing pain or impeding breathing. The final Belt design together with the mold and assembly are shown in Supplementary Figure S5.

### 3.3 Second cohort trial: third Bib design

According to the testing protocol, ten mice (MAW 20.3 ± 2.51 g) were dressed with the Simple Bib design with only the MH-holder (Supplementary Figure S6A) and the Bib (Supplementary Figure S6B): four mice with the MH containing the magnets and six with the mock MH containing only lead weights. The Bib size was adjusted to the mouse’s weight and size (Supplementary Figure S6B). All animals immediately expressed the clear intention of getting rid of the device once mounted, with one mouse managing to remove the device (it was immediately dressed again with a smaller Bib). After approximately 40 min, none were still trying to remove the device (Figure 4C). Five mice (MAW 20.4 ± 3.06 g) were dressed with the Slide Bib concept. Once awakened, they showed fatigue in carrying the backpack, initially flipping over on their backs and being unable to regain the correct “paws down” position; however, after 20 min of habituation, they were able to handle the weight and avoid flipping. Their condition was controlled for the 7 days of the habituation period. Eight Simple Bib animals kept the Bib prototypes and proceeded to the injury step. Two Slide Bib mice, on the other hand, showed altered behaviors starting at day 3. Two mice were undressed, and only three reached the injury step. After the injuries, eight mice were dressed with the Simple Bib and three with the Slide Bib.

For the Simple Bib design, all injured mice, once awakened, showed fatigue in carrying this device, flipping over onto their backs and struggling to straighten up. After 30 min, they all managed to deal with the device’s weight and were able to flip back autonomously. After 1 h, they could all balance themselves and reduce flipping-over events while moving in the cage. On day 3, three animals were again incapable of flipping back autonomously, so they were undressed. After week 1, three of the five mice still carrying the device manifested incomplete grooming, lower appetite, and general distress, so they were all undressed. Only two mice kept the Bib on for 14 days, and they did not complete the required 3 weeks.

For the Slide Bib design, all the injured mice, once awakened, manifested fatigue carrying the device, flipping over on their backs, and struggling to straighten up. Only two mice, after 1 h, managed to deal with the device’s weight and were able to flip back autonomously, while the remaining mouse was undressed. Both mice kept the Slide Bib on for 1 week. During the second week, one mouse showed altered behavior (reduced grooming and feeding) and was undressed; the remaining mouse was undressed at the beginning of the third week without completing the required period.

Both solutions, the Simple Bib and Slide Bib, also underlined that excessively tightening the device around the animals’ trunk compromised their natural propensity for activities like nutrition and grooming. An additional limitation is correlated with the necessity of maintaining at least two animals per cage due to their social nature: two or more mice together in a cage tried to free each other by biting the Bib. The social nature and general biting attitude of mice enhanced cooperative behaviors of biting the silicone-based structure to free their cage-mates.

### 3.4 Fourth design: the Cape

In this last trial, as described in “Methods”, a split habituation period was included. The Bib trial suggested the need for reducing animal stress from adding both a tight dress and the MH weight. In this trial, the final and lightest MH design was used (Figure 3D). The Cape, with its reduced thickness and tighter fit to the mouse body, is shown in Figure 4D (left), and it reduced the tendency of the animals to bite the device while increasing compliance for movement and breathing. Thanks to the habituation period, the mice had more time to become familiarized with the new attachment on their bodies before the application of the magnets’ weight (Figure 4D, center and right).

Five mice, (MAW 20.4 ± 1.14 g) were dressed with the Cape; after 3 days of habituation, the MH weight was glued to their back without needing additional anesthesia. All mice quit trying to undress after a few (<5) minutes from awakening; after the three habituation days, they were perfectly comfortable with the Cape. When the MH weight was applied, they all handled it perfect—no flipping episodes were recorded, and no signs of impaired feeding or grooming were detected. Therefore, they all proceeded to the injury step.

After injury, all five mice were dressed with the Cape; their behavior after the Cape dressing reflected what was already observed during the habituation period. No flipping episodes were recorded, and wellness scoring was always good for 14 days. Not all mice reached week 3 (two mice were undressed for reasons unrelated to the device). The remaining three mice successfully reached the end of week 3 (Figure 4D). They were all undressed and a fur check confirmed that the Cape had not caused skin maceration.

#### 3.4.1 Prototype comparison

**FIGURE 7:**
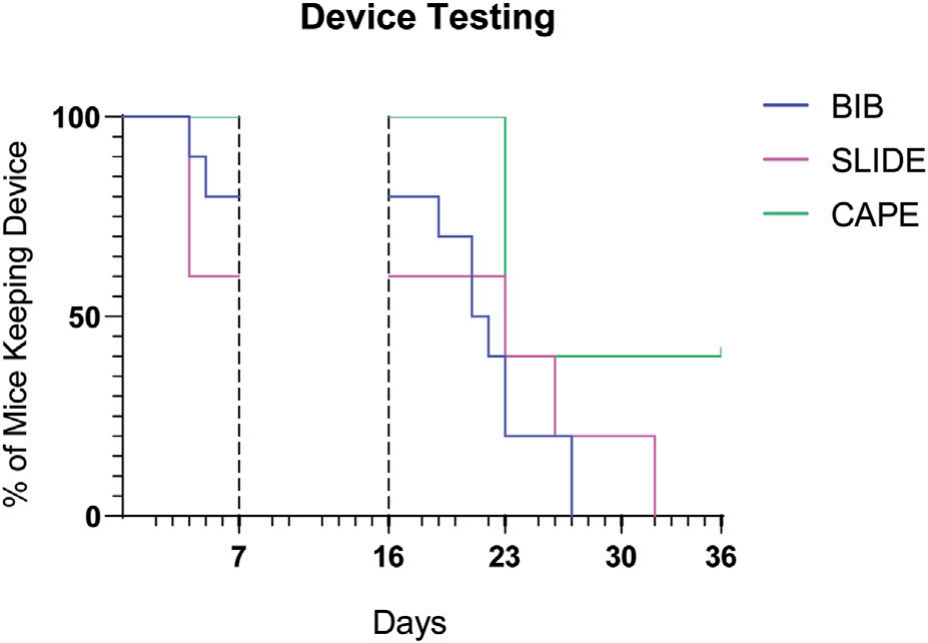
Prototypes compared over time. Kaplan–Meier graph illustrates, for each prototype, the percentage of mice wearing the device over time. The time periods shown here correspond to the protocol phases (habituation period and wearable device application after injury and transplant), as described in Figure 5. Mice were removed from trial if i) they spontaneously undressed or ii) the operator was forced to undress them in case of stress or distress signs.

Figure 7 depicts the results of the Kaplan–Meier maintenance analysis with the outcomes of various designs tested in the clinical trial setting. Among the designs evaluated, the Cape design, which underwent testing in the third cohort trial, exhibited the most promising rates of being worn over time. This finding underscores the potential efficacy and durability of the Cape design. The Kaplan–Meier maintenance analysis, coupled with the log-rank test, provides compelling evidence supporting the superiority of the Cape design (*p*-value of 0.01).

One additional aspect to consider is the total weight of the wearable device. The final, accepted Cape prototype weighed 7.98 g (including magnets) compared to the 10.18 g total weight of the heaviest Slide Bib. The overall MAW of the enrolled animal was 20.43 ± 2.04 g.

## 4 Discussion

Our design and prototyping efforts have led to the development of a novel wearable device, fabricated through 3D printing technology, tailored for mice, and specifically designed for the non-invasive, long-term application of external devices that need to maintain continuous contact with the dorsal region of the animal at a fixed location.

Considering the variability in the body weight of healthy or injured mice aged 3–16 weeks and the ensuing differences in their potential carrying capability, we designed and evaluated various configurations. In this process, we reasoned that the use of rigid materials can affect the behavior of injured animals much more than flexible and compliant materials suitable for holding the magnets. Lastly, to accommodate the changing dimensions of the bodies of growing animals during prolonged stimulations, this material allows the creation of a fully personalized wearable apparatus.

The iterative design process addressed various challenges and considerations, ultimately achieving a balance between wearability, stability, and the correct promotion of axonal growth. The evolution through different design iterations, such as the abandoned Fitbit, problematic Belt, challenging Bib, and cumbersome Slide Bib designs, provided valuable insights into the limitations and challenges faced during the development process. In contrast with previous designs which exhibited limitations, such as the Bib, resulting in altered behaviors, reduced grooming, and compromised nutrition, the Cape design, with its reduced thickness and increased adherence, addressed the shortcomings observed in previous iterations. The incorporation of a split habituation period proved pivotal in acclimating mice to the new wearable device, thus fostering compliance and minimizing stress. The testing protocol, encompassing evaluations of both healthy and injured mice, ensured a comprehensive assessment of wearability and its impact on animal welfare. Notably, the Cape design performed remarkably, with mice comfortably keeping the device in place for at least 21 consecutive days. In contrast, previous designs such as the Bib exhibited limitations, resulting in altered behaviors, reduced grooming, and compromised feeding.

In our investigation of the weight-bearing capacity of mice, we initially referred to existing literature indicating that mice can typically bear at least their own weight—although with limited studies available, notably Deacon (2013)—and that continuous applied weight could reach 10 g for 6–8-week-old mice (Shekhtmeyster et al., 2023). However, there seem to be no trials that have specifically examined continuous weight application. Our experience leads us to conclude that a tolerable weight for both healthy and injured mice is approximately one-third of their body weight. This observation calls for potential studies exclusively dedicated to weight application to systematically analyze this important aspect of wearable device set-up.

Our study confirms the opportunity offered by 3D printing in the field of customized designs for animal testing. Moreover, it not only allows the successful generation of wearable solutions for the mechanical stimulation of spinal cord injury (SCI) mice but also provides valuable guidelines for researchers and practitioners involved in testing other stimulation strategies for preclinical studies on experimental models of SCI. Indeed, different stimulation-based therapeutic approaches for SCI are currently being tested, including electrical spinal cord stimulation (Seáñez and Capogrosso, 2021) trans-spinal magnetic stimulation (Chalfouh et al., 2020), and the use of exoskeletons (Mekki et al., 2018). However, their preclinical validation is severely hindered by the lack of strategies for generating wearable devices that mimic their human counterparts by simultaneously addressing mouse model specificity in terms of ergonomics and tolerability. The development of wearable devices for SCI models will certainly improve the translational potential of these studies, eliminating the animal distress caused by traditional invasive approaches. The emphasis on non-invasive approaches and the consideration of animal welfare throughout the design process make our findings relevant and applicable in the broader scientific context of long-term wearable devices.

Our work may open new avenues for advancing the field of wearable devices in preclinical studies, particularly in the context of research on new and fully effective therapies for SCI.

## 5 Conclusion

Our research presents a significant advance in the development of wearable devices for mice—specifically, for non-invasive, long-term applications requiring continuous dorsal contact. Through iterative design and prototyping efforts, we have successfully created an ideal device, the “Cape”, fabricated with a combination of molding and 3D printing technology. Our study not only demonstrates the feasibility of 3D printing in creating customized solutions for animal testing but also provides valuable insights into the design considerations necessary for promoting animal welfare and compliance. Our work goes beyond the specific application of spinal cord injury models, offering guidelines that are useful in the broader range of preclinical studies involving wearable devices.

## Data Availability

The raw data supporting the conclusion of this article will be made available by the authors, without undue reservation.
