# Supplementary material for: 3D-printed weight holders design and testing in mouse models of spinal cord injury
Source: Front Drug Deliv. 2024 May 22;4:1397056. doi: 10.3389/fddev.2024.1397056 (PMC12363281; doi:10.3389/fddev.2024.1397056)
Supplement: Supplementary file 1 [file DataSheet1.PDF]

Supplementary material.

3D printed weight holders design and testing in mouse models of spinal cord injury

Sara De Vincentiis, Francesca Merighi, Peter Blümler, Jose Gustavo De La Ossa Guerra, Maria Chiara Di Caprio, Marco Onorati, Marco Mainardi, Vittoria Raffa , Marina Carbone

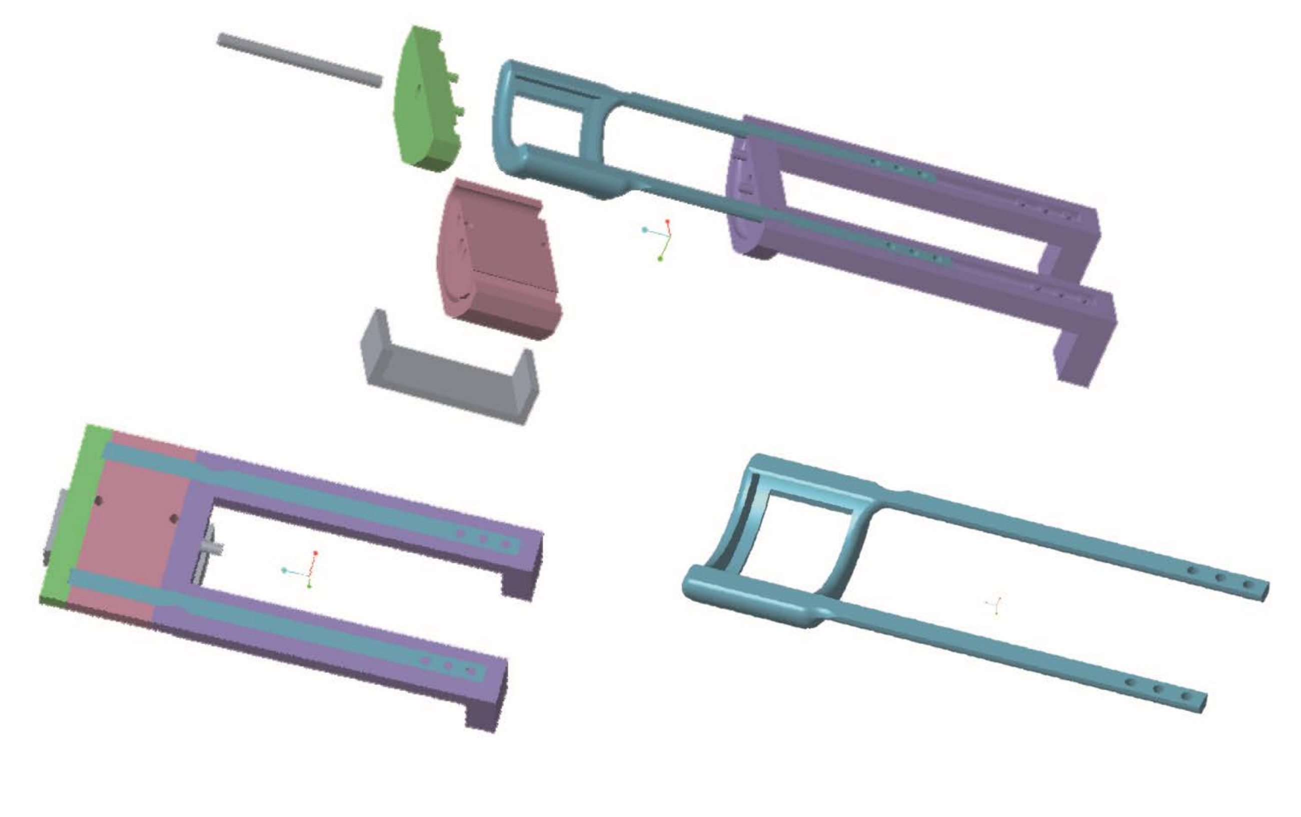

Figure S 1 The "Fitbit" approach. Mold design for the wraparound component ("Fitbit" band) and the two suspenders. The mold is composed of five components which leave the negative form of the desired object (light blue). The "Fitbit" is obtained from pouring silicone into the negative form and demolding it after curing.

Supplementary material.

3D printed weight holders design and testing in mouse models of spinal cord injury

Sara De Vincentiis, Francesca Merighi, Peter Blümler, Jose Gustavo De La Ossa Guerra, Maria Chiara Di Caprio, Marco Onorati, Marco Mainardi, Vittoria Raffa , Marina Carbone

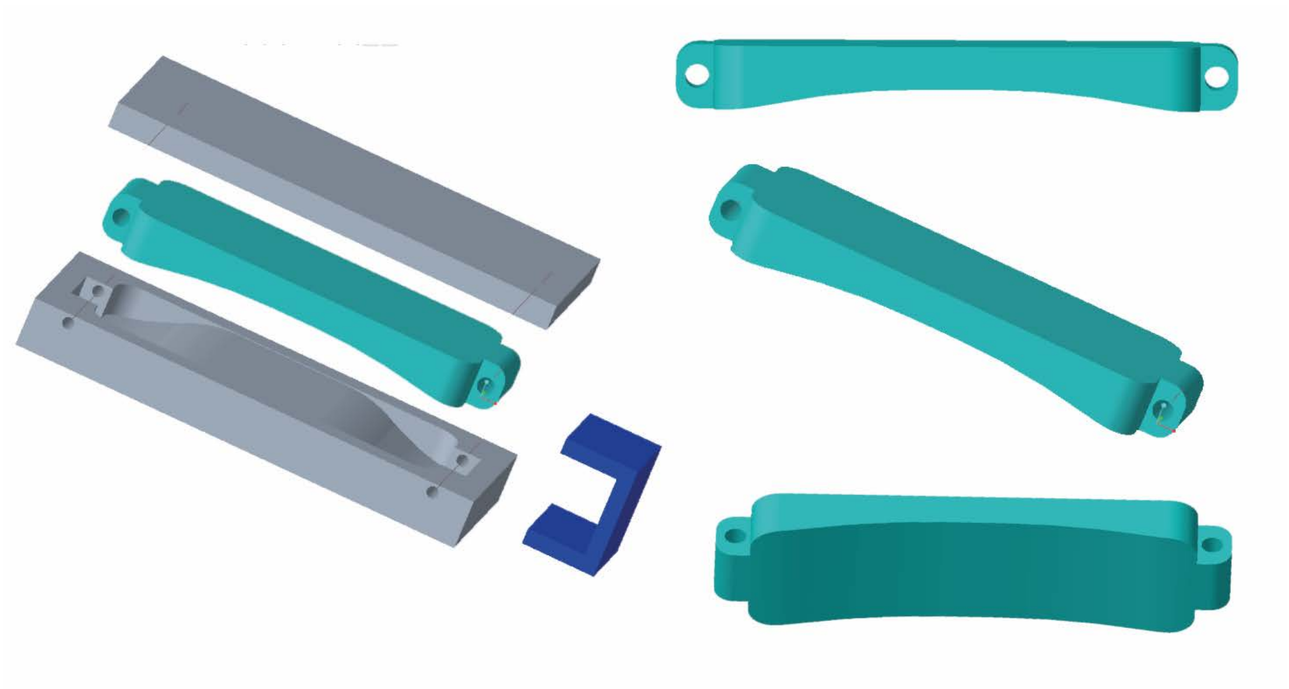

Figure S 2 The "Belt" approach. On the left: the design of the mold (gray and blue) and a representative image of the soft silicone component (turquoise) of the "Belt". The mold is composed of two main elements that should be assembled as shown. The silicone is poured into the void between them and after curing the "Belt"'s strap is formed. On the right: the strap from different perspectives.

Supplementary material.

3D printed weight holders design and testing in mouse models of spinal cord injury

Sara De Vincentiis, Francesca Merighi, Peter Blümler, Jose Gustavo De La Ossa Guerra, Maria Chiara Di Caprio, Marco Onorati, Marco Mainardi, Vittoria Raffa , Marina Carbone

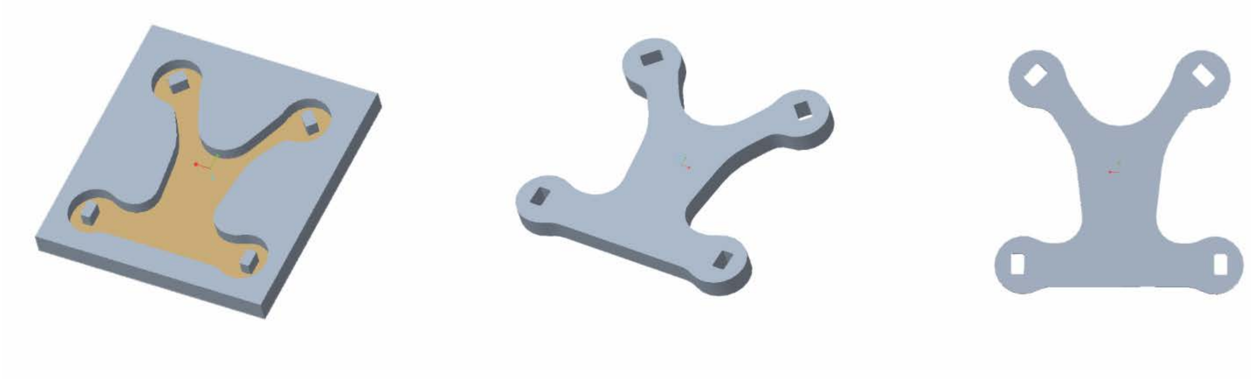

Figure S 3 ***The "Bib" approach.*** *(Left: design of the mold of the "Bib". It is composed of one single element with a pit into which silicone is poured and allowed to cure. In the middle and on the right: representative images of the "Bib".*

Supplementary material.

3D printed weight holders design and testing in mouse models of spinal cord injury

Sara De Vincentiis, Francesca Merighi, Peter Blümler, Jose Gustavo De La Ossa Guerra, Maria Chiara Di Caprio, Marco Onorati, Marco Mainardi, Vittoria Raffa , Marina Carbone

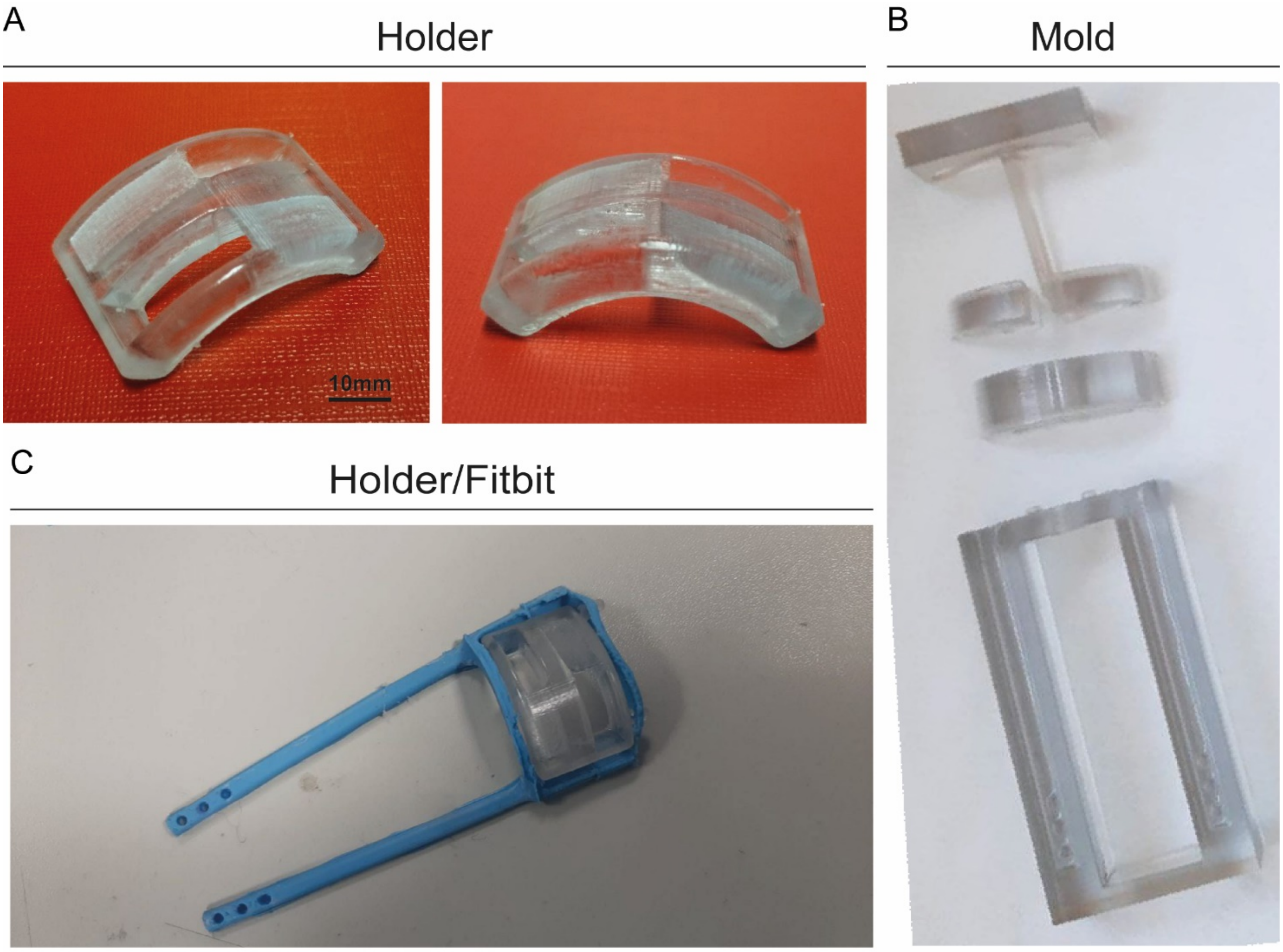

Figure S 4 The "Fitbit" approach. (A) Representative pictures of the first design of MH after 3D printing. The magnets are placed in the holes. (B) Representative picture of the mold for the "Fitbit" after the 3D printing. (C) Representative picture of an assembled 3D printed MH and silicone "Fitbit".

Supplementary material.

3D printed weight holders design and testing in mouse models of spinal cord injury

Sara De Vincentiis, Francesca Merighi, Peter Blümler, Jose Gustavo De La Ossa Guerra, Maria Chiara Di Caprio, Marco Onorati, Marco Mainardi, Vittoria Raffa , Marina Carbone

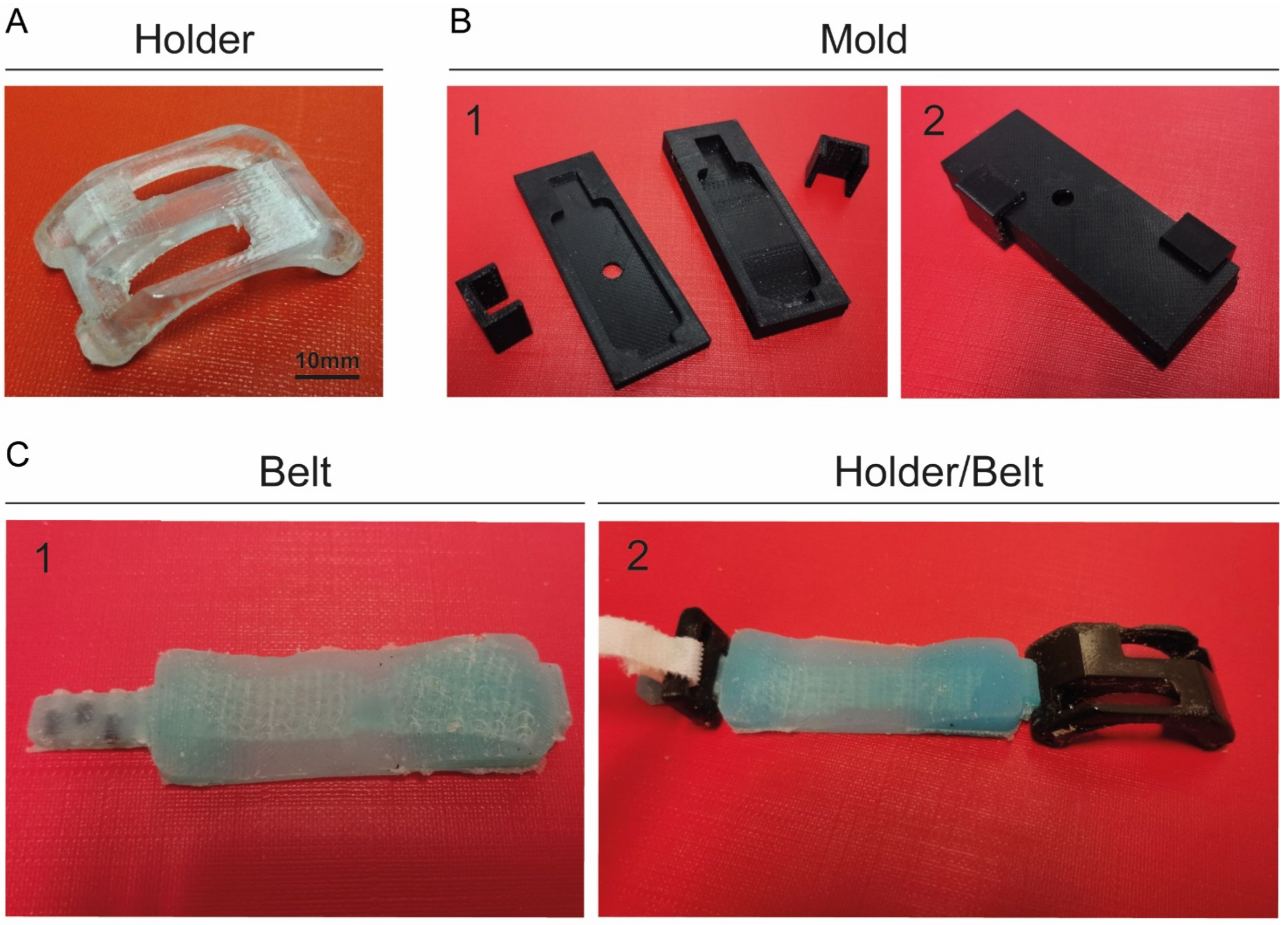

Figure S 5 The “Belt” approach. (A) Representative pictures of the 3D printed MH second design. The holder is modified by a cut on the superior surface. (B) Representative picture of the mold for the soft part of the “Belt” after 3D printing. The mold is shown in open configuration (B1) with all the components separated; in B2, the mold is shown in the closed configuration, ready for adding silicone. (C) Pictures representing all the elements that compose the “Belt”: in C1 the soft component is shown, obtained from a silicone setting inside the mold. C2 displays the complete “Belt”, with the rigid components attached to the soft one.

Supplementary material.

3D printed weight holders design and testing in mouse models of spinal cord injury

Sara De Vincentiis, Francesca Merighi, Peter Blümler, Jose Gustavo De La Ossa Guerra, Maria Chiara Di Caprio, Marco Onorati, Marco Mainardi, Vittoria Raffa , Marina Carbone

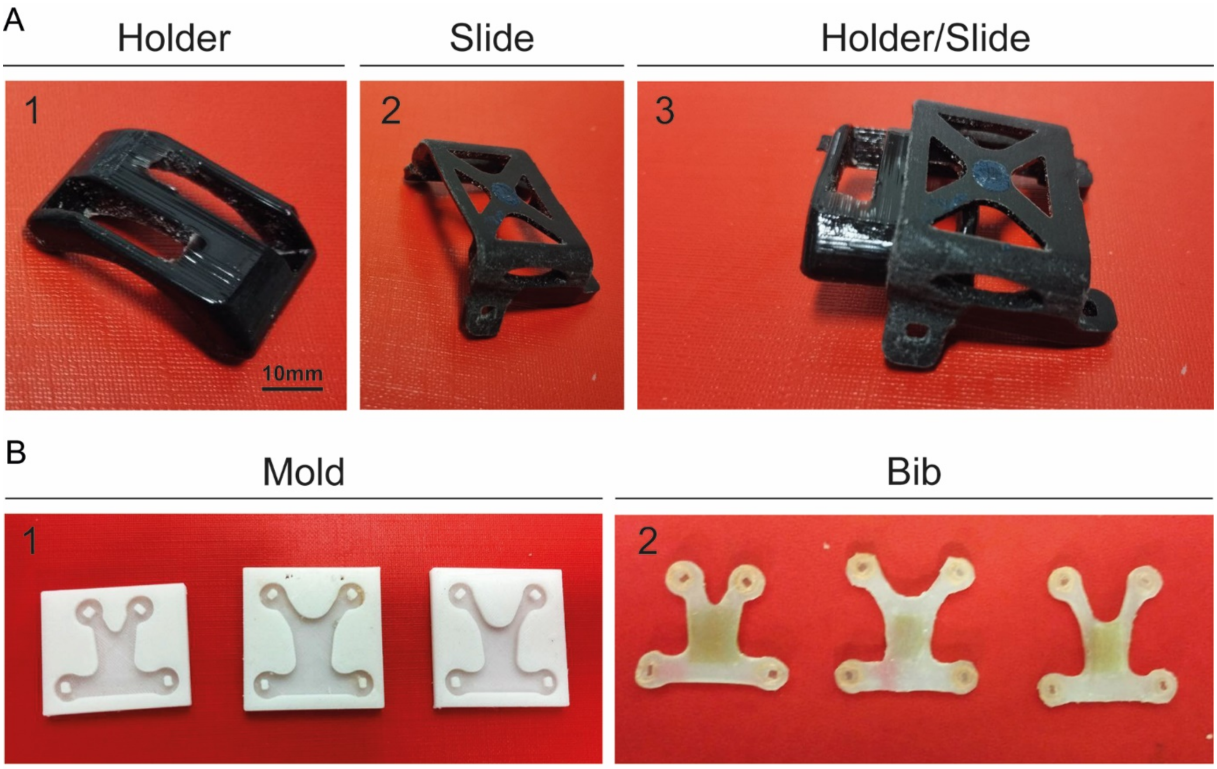

Figure S 6 13 The "Bib" approach. (A) Representative pictures of the 3D printed MH second design (1), of the slide (2) and the MH and slide assembled together (3). (B) Representative pictures of three molds for the „Bib" after 3D printing. The shown molds differ in length and distance between the two anterior suspenders (1). On the right, representative pictures of the „Bib" obtained from the respective mold shown in B1. (C) Pictures representing all elements that compose the „Bib" assembled together. C1 shows the MH outside and the „Bib" fastened to the slide. In C2 the insertion of the MH in the slide is displayed. In C3 the assembled structure is shown from below, showing how the „Bib" is fastened to the slide with cable ties.
